# TIPsy tour guides: how microtubule plus-end tracking proteins (+TIPs) facilitate axon guidance

**DOI:** 10.3389/fncel.2015.00241

**Published:** 2015-06-30

**Authors:** Elizabeth A. Bearce, Burcu Erdogan, Laura Anne Lowery

**Affiliations:** Department of Biology, Boston CollegeChestnut Hill, MA, USA

**Keywords:** +TIPs, axon guidance, growth cone, microtubule dynamics, cytoskeleton

## Abstract

The growth cone is a dynamic cytoskeletal vehicle, which drives the end of a developing axon. It serves to interpret and navigate through the complex landscape and guidance cues of the early nervous system. The growth cone’s distinctive cytoskeletal organization offers a fascinating platform to study how extracellular cues can be translated into mechanical outgrowth and turning behaviors. While many studies of cell motility highlight the importance of actin networks in signaling, adhesion, and propulsion, both seminal and emerging works in the field have highlighted a unique and necessary role for microtubules (MTs) in growth cone navigation. Here, we focus on the role of singular pioneer MTs, which extend into the growth cone periphery and are regulated by a diverse family of microtubule plus-end tracking proteins (+TIPs). These +TIPs accumulate at the dynamic ends of MTs, where they are well-positioned to encounter and respond to key signaling events downstream of guidance receptors, catalyzing immediate changes in microtubule stability and actin cross-talk, that facilitate both axonal outgrowth and turning events.

## Introduction

**Figure 1:**
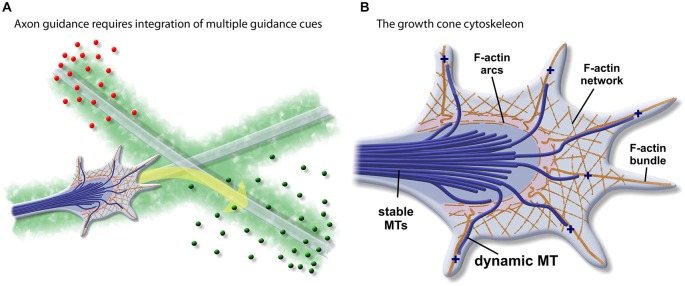
**The growth cone cytoskeleton.** **(A)** The axon has to travel a complex landscape which presents a multitude of external chemotropic cues (e.g., red and green circles represent diffusible cues, light green represents substrate-bound cues). To properly interpret and navigate these signals, the axon is equipped with a dynamic cytoskeletal vehicle called the growth cone. **(B)** Structural organization of the growth cone cytoskeleton: Bundled, stable Microtubules (MTs) extend through the axon, entering the growth cone “wrist.” These terminate in the growth cone “central” domain, surrounded by a cage of F-actin arcs. F-actin bundles extend into the growth cone “fingers” (filopodia) to sample the environment. Between the filopodia there exist a series of cortical actin networks which create the lamellipodial veils. A subset of MTs can escape the central domain, to trace along F-actin structures and explore the growth cone periphery. The dynamic plus-ends of MTs are decorated by set of proteins called plus-end tracking proteins (+TIPs) (not shown). Concentration of +TIPs at the dynamic leading edge is particularly important as they come in close contact with signaling cascades that are triggered by external cues.

The young neuron faces a complex journey. Extending a single, properly guided axon through the developing embryo involves intricate orchestration of growth, retraction, and turning events. To navigate this landscape, the axon is equipped with a responsive cytoskeletal vehicle called the growth cone, which uses a well-organized network of actin filaments to maneuver and power forward. In order to choose the right course, the axon is presented with a multitude of external, chemotropic cues, which must be detected and then effectively translated into an appropriate mechanical response (Figure 1A). The spatially-restricted stabilization of microtubules (MTs) has long been established to be integral for turning behaviors. Evidence has emerged only more recently that suggests that their plus-end tracking proteins (+TIPs) intercede as signal transduction “tour guides” during axon guidance. Utilizing their capacity to interface with both microtubule and actin cytoskeletons, as well as their close-proximity to the cortical guidance cascades, +TIPs are well-positioned to inform and direct growth cone behaviors. In this review, we highlight seminal and current works on +TIP-mediated signal transduction and regulation of cytoskeletal dynamics in axon elongation and turning events.

### The Growth Cone Cytoskeletal Vehicle

The basic organization of the growth cone cytoskeleton has been well-characterized (Geraldo and Gordon-Weeks, 2009; Lowery and Van Vactor, 2009; Dent et al., 2011; Hur et al., 2012; Figure 1B). The bundled MTs of the axon shaft give way to the contractile actomyosin “wrist” at the base of the growth cone. In the growth cone central domain, stable MTs are corralled into a dense cluster by actin arcs. Filamentous (F)-actin bundles extend into the growth cone periphery, forming the filopodia “fingers.” Cortical actin networks surround the filopodia, forming lamellipodia-like veils. Singular dynamic, pioneer MTs escape the central domain with their dynamic “plus-ends” oriented towards the filopodial tips. These MTs transiently couple to the F-actin-based filopodial tracks, allowing them to explore the outer reaches of the actin cytoskeleton (Schaefer et al., 2002). The positioning of these MTs within the growth cone periphery allows them to aid in vesicle transport, couple to actin networks, and encounter signaling molecules.

### Microtubules in Axon Outgrowth and Turning

Both actin and MTs play a critical role in coordinated growth cone motility and steering. It is generally accepted that F-actin networks, by way of F-actin treadmilling and adhesion dynamics, provide protrusion and motility to the growth cone vehicle (Dent et al., 2011). However, early studies of growth cone dynamics noted that singular MTs were capable of escaping the central growth cone domain, and that the orientation of these MTs often predicted the direction of subsequent outgrowth (Sabry et al., 1991; Tanaka et al., 1995). When focus was drawn to these “pioneer” MTs of the growth cone periphery, it became evident that their selective stabilization could impact growth cone navigation events. Localized application of the microtubule stabilizing drug, Taxol, was sufficient to induce growth cone turning towards the site of administration, whereas the opposite turning effect was seen with administration of a MT-depolymerizing drug, Nocodazole (Buck and Zheng, 2002). Attractive turning could be extinguished by subsequent inhibition of either actin or Rho GTPases, indicating that this initial stabilization of MTs precedes and facilitates actin remodeling, potentially through subsequent activation of Rho GTPases (Buck and Zheng, 2002). While these studies highlighted an important role for pioneer MTs in axon outgrowth and turning events, thus establishing MTs as important navigators of the growth cone vehicle, it was not immediately clear how biologically-relevant extracellular guidance cues might result in targeted, downstream changes in growth cone MT stability. However, the more general question of how MTs are stabilized, destabilized, or otherwise modulated has long been a topic of interest in many biological contexts.

### +TIPs Decorate the Ends of MTs and Contribute to their Dynamic Instability

MTs are subject to regulation by a multitude of MT associated proteins (MAPs), which act to nucleate, stabilize, destabilize, sever, bundle, or additionally modify MT behaviors (Andersen, 2000; Akhmanova and Steinmetz, 2008). A subset of these, which selectively ride along, or “track” the dynamic MT plus-end, are appropriately-named the +TIPs (Akhmanova and Steinmetz, 2008). But +TIPs are not passive hitchhikers; rather, they are themselves dynamic and interactive, undergoing phosphorylation and other modifications which can induce MT-actin crosslinking, interactions with focal adhesion scaffolding, or rapid dissociation from the plus-end. Their malleable behaviors make them ideal “first responders” within axon guidance pathways, serving as informative translators along the journey. Here, we present a collection of evidence to substantiate a role for +TIPs as molecular tour guides during axon elongation and turning events.

## The +TIP of the Iceberg: Cytoplasmic Linker Associated Protein (CLASP) and Adenomatous Polyposis Coli (APC) Emerge as Axon Guidance Modulators

**Figure 2:**
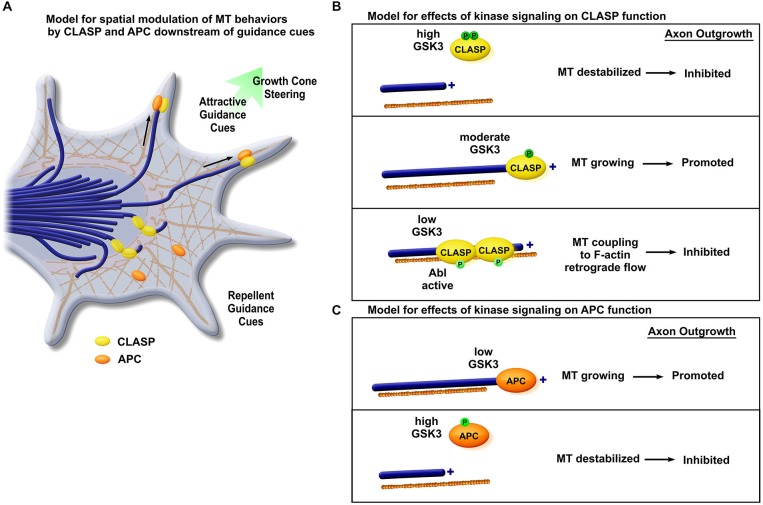
**Phosphorylation dependent +TIPs spatial distribution along MT is key to axon outgrowth and steering.** **(A)** Spatial distribution of CLASP and APC is key to the modulation of MT dynamic behavior and generation of the navigational response. Asymmetrically-distributed guidance signals differentially regulate +TIP localization. On the side of attractive guidance cues, CLASP and APC demonstrate MT plus-end binding and promote axon outgrowth. Faced with repellent cues, APC dissociates from MTs and CLASP shows lattice binding, inhibiting axon outgrowth. **(B)** Distribution of CLASP is modulated by phosphorylation by kinases. High levels of GSK3 activity lead to two sites of phosphorylation of CLASP, which then dissociates from MTs causing MT destabilization and axon outgrowth inhibition (upper panel). Moderate GSK3 activity promotes a single site of phosphorylation of CLASP, facilitating CLASP plus-end localization and axon growth (middle panel). With low levels of GSK3, CLASP remains unphosphorylated, and CLASP binds to the MT lattice, inhibiting axon growth by coupling MTs to F-actin retrograde flow. A similar phenotype is observed when CLASP is exposed to high levels of Abl activity, which leads to phosphorylation at a different site (lower panel). **(C)** Phosphorylation-dependent APC distribution. Low GSK3 activity allows APC to remain unphosphorylated, promoting its association with plus-ends, facilitating axon growth (upper panel). However, under high GSK3 activity, phosphorylated APC dissociates from MTs and causes MT destabilization of MTs and axon growth inhibition (lower panel).

The first formative links between MT plus-end regulation and extracellular signaling cues were forged when two disparate +TIPs were simultaneously shown to possess distinct roles in relaying information downstream of axon guidance signaling events (Figure 2). Utilizing an elegant, *in vivo* genetic screen in the *Drosophila* retina, Lee et al. showed that *mast/orbit*, an ortholog to the MAP, cytoplasmic linker associated protein 1 (CLASP 1), cooperates with Abelson tyrosine kinase (*Abl*), a downstream signaling molecule involved in the Slit repellent pathway (Lee et al., 2004). This genetic interaction was fortified when *mast/orbit* and *Abl* zygotic loss-of-function mutants displayed an identical ectopic central nervous system midline crossing phenotype and similar deficits in motor axon pathfinding within the peripheral nervous system. To gain a mechanistic understanding of the function of CLASP within growth cones, and to elucidate why manipulation of this +TIP might result in an axon guidance phenotype, GFP-CLASP was expressed in growth cones of *Xenopus laevis* (an ideal system for imaging cytoskeletal dynamics due to their large size and ease of neuronal culturing (Stout et al., 2014)). CLASP was found to localize to growing MT plus-ends specifically within the growth cone and preferentially track the ends of pioneer MTs which ran along actin filopodia, where CLASP would theoretically be exposed to Abl signaling cues. Overexpression of CLASP, however, demonstrated severe central-zone MT looping, where MTs were unable to escape into the growth cone periphery, and growth cone advance was significantly reduced. Together, these data provided strong evidence that CLASP was implicated downstream of Abl kinase signaling events to regulate growth cone MT dynamics.

Separately, Zhou et al. provided evidence that interactions between pioneer MTs and the +TIP APC were facilitated downstream of the axonal outgrowth signaling molecule nerve growth factor (NGF) in murine dorsal root ganglion neurons (Zhou et al., 2004; Figure 2). This was the first record of a role for APC in the nervous system, though its plus-end localization had previously been shown in other cell types (Zumbrunn et al., 2001). Here, APC was shown to localize strongly to MT plus-ends in growth cones. However, this association could be reduced if NGF was removed, leading to the hypothesis that APC bound to plus-ends in response to localized inactivation of one of NGF’s downstream signaling effectors, GSK3. This was tested with numerous pharmacological inhibitors and mutant constructs, which verified that APC operated directly downstream of GSK3b to locally-stabilize MTs when GSK3 was inactive. This selective stabilization occurred simultaneously with actin remodeling. Together, these data supported a model for APC promoting axonal elongation, downstream of signaling cues.

### Expanded Roles of CLASP: Navigating Downstream of Two Kinases

Subsequent explorations of CLASP’s role in axon outgrowth and guidance have expanded details of its regulation to include modulation not only by Abl, but by GSK3, as well. While significant progress has been made in understanding the interplay between these two guidance cascades, early studies examining the role of CLASP on a cellular level initially painted a muddled picture.

First, it was not exceedingly clear what role CLASP played in axon elongation. Lee et al. (2004) associated it genetically with Slit, and consequently, with repulsive turning events. However, CLASP was already known to be a MT stabilizing protein in multiple systems (Lemos et al., 2000; Akhmanova et al., 2001; Galjart, 2005). Therefore, a role in repulsive turning seemed to go against seminal observations that localized MT-stabilization would promote outgrowth and attractive turning (Buck and Zheng, 2002). When CLASP was then found to be a substrate of GSK3, this added another layer of complexity; GSK3 had been implicated in both promoting and inhibiting axon elongation (Owen and Gordon-Weeks, 2003; Zhou et al., 2004). Solutions to these controversies were somewhat intertwined, as later work would reveal that CLASP was not solely a +TIP, but additionally possessed a MT lattice-binding activity. Signaling events downstream of guidance cues, through GSK3 and Abl, could control the switch between these two modes of MT interaction.

When Hur et al. (2011) examined GSK3 phosphorylation of CLASP in the growth cone, they showed that rather than complete GSK3 inactivation, a balance of GSK3 inhibition was necessary to allow axon outgrowth. Thus, an understandable model finally emerged (Hur et al., 2011; Figure 2). There were two separate sites available for phosphorylation of CLASP by GSK3. In cases of highly-active GSK3, both sites were phosphorylated, CLASP dissociated from MTs, and axon outgrowth was reduced. Where only one site was phosphorylated, plus-end binding was optimized, promoting MT stability and axon elongation. However, when GSK3 was entirely inactive, CLASP remained dephosphorylated at these sites, which enabled strong association to the MT lattice. MTs then accumulated within the central growth cone, and axon outgrowth was stunted. This phenotype could be largely rescued by the myosin-II inhibitor blebbistatin, indicating that MTs were being excessively-coupled to F-actin retrograde flow. These findings in neurons were consistent with previous studies of CLASP regulation by GSK3 in non-neuronal cells (Akhmanova et al., 2001; Wittmann and Waterman-Storer, 2005; Kumar et al., 2009, 2012). Separately, CLASP depletion in the growth cone was also shown to strongly perturb actin organization, resulting in collapsed, weakened F-actin architecture within lamellipodial veils (Marx et al., 2013), supporting an idea that balanced CLASP-plus-end and CLASP-lattice (and F-actin) interactions are necessary for optimal growth cone structural integrity.

Subsequent biochemical studies of CLASP phosphorylation by Abl demonstrated similar functional interactions, in that Abl phosphorylation also occurred within a domain that mediated both MT and actin binding, and was thus also able to “toggle” CLASP behavior (Engel et al., 2014). Overexpression of constitutively-active Abl resulted in a phenotype that mirrored GSK3 inhibition, with decreased CLASP localization at MT plus-ends, accumulation of CLASP with actin-rich structures in the central growth cone domain, and growth cone pausing (Engel et al., 2014). While it was already well-established that Abl signaling regulates F-actin organization by Ena/VASP (Gertler et al., 1995; Wills et al., 1999; Bashaw et al., 2000; Lin et al., 2009), this work suggests that Abl regulation of CLASP may also affect F-actin organization, once again highlighting the complex interplay at work within the growth cone cytoskeleton.

### APC: A Jack of all Trades, and Putative Growth Cone Synthesis Hub

Similar to CLASP, APC was established as a +TIP which was thought to stabilize MTs even prior to its implications in axon guidance, and it had already been speculated that its phosphorylation state may affect its association to MTs (Smith et al., 1994; Su et al., 1995; White, 1997; Zumbrunn et al., 2001). Like other plus-end stabilizing proteins, APC was found to be greatly enriched in the nervous system during axon growth and navigation (Bhat et al., 1994; Koester et al., 2007). A series of works emerged that pointed to GSK3-modulated APC in multiple roles within the early nervous system, including neuronal polarity, migration, and axon specification, providing some added assurance that this interaction was an important regulator of MT dynamics in neural development (Dobashi et al., 2000; Shi et al., 2004; Koester et al., 2007; Purro et al., 2008; Eom et al., 2014; Mohn et al., 2014; Onouchi et al., 2014). APC preferential localization was shown to accumulate on the “turning” side of the growth cone as it encountered a substrate border, predicting turning behavior (Koester et al., 2007; Figure 2). Evidence in mouse neurons also pointed towards a role for APC downstream of Wnt signaling, as growth cone pausing, MT looping, and reduction in APC plus-end localization can also be induced by Wnt3a (Purro et al., 2008). But this newer function in axon guidance (Zhou et al., 2004) did not come without discourse: the dominant negative strategies that first identified APC as a candidate for axon guidance in murine models met some critique when complete ablation of the APC ortholog in *Drosophila* neuronal precursors caused perceptible effects on polarity and axon outgrowth of some but not all neurons (Rusan et al., 2008).

However, recent findings pointing to a role for APC in translation might explain the discrepancy between these previous experiments. APC gained new acclaim as strong evidence emerged that it serves not only as a MT-effector, but also as an RNA-binding protein in neurons (Preitner et al., 2014). A HITS-CLIP interactome generated from RNA targets of APC revealed a large pool of highly-interrelated cytoskeletal mRNAs, opening up the provocative possibility that APC may sequester localized cytoskeletal protein factories, an idea supported by demonstration of its ability to facilitate local synthesis of an axon-specific tubulin isotype, beta2B-tubulin (Preitner et al., 2014). This is especially intriguing when taken into consideration with evidence that APC contains a region on its N-terminal that permits self-association and clustering (Li et al., 2008), which could potentially greatly amplify the amount of mRNA sequestered in one peripheral region. The capacity of APC to localize mRNA could explain the discrepancy noted between earlier murine and *Drosophila* studies of plus-end function and axon outgrowth, as the *Drosophila* studies deleted APC, while the murine experiments disrupted APC-MT plus-end interactions without ablating it, potentially contributing to mislocalization of APC-interacting mRNAs. This mRNA-localization function is thus far unique among +TIPs, but represents an enticing possibility that some members of the family could not only modulate turning events, but also direct local translation and thereby facilitate rapid outgrowth from these regions of stabilized, pioneer MTs. This possibility is further bolstered by the fact that a screen for CLASP interactors identified multiple RNA processing factors (Lowery et al., 2010), although a direct role for CLASP in translation regulation remains to be seen.

## A New Team of Tour Guides: An Expanding Family of Tip-trackers Shows Promise as Regulators of Axon Guidance

**Figure 3:**
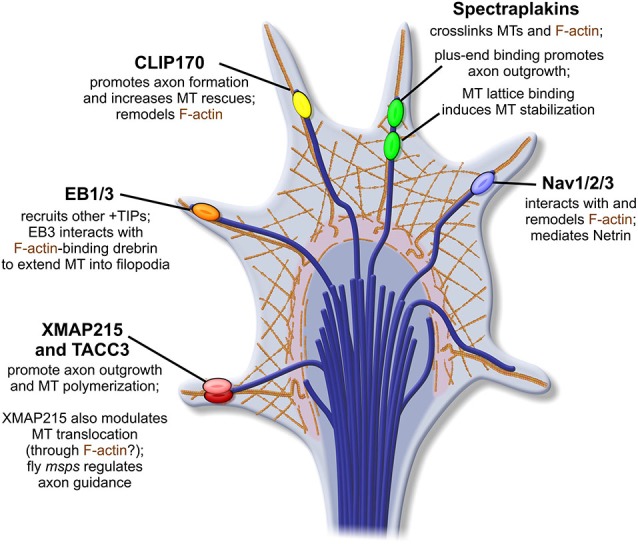
**Additional +TIPs in axon outgrowth and guidance.** A brief synopsis of other presently-identified +TIPs that function in axon outgrowth and guidance, highlighting some of their known interactions: note that a number of these proteins demonstrate an ability to facilitate MT-F-actin coupling.

The initial explorations on CLASP and APC paved the way for further genetic and cell biological approaches investigating +TIP function within the growth cone. These studies began to quickly reveal a growing cast of +TIPs that contribute far beyond their prescribed roles in MT stability. It is now apparent that their functions include coupling MT dynamics to F-actin networks, promoting or inhibiting axonal outgrowth, and facilitating growth cone turning, as described in more detail below (Figure 3).

### XMAP215

XMAP215 was initially identified as a MT-growth enhancing protein isolated from *Xenopus* egg extracts (Gard and Kirschner, 1987), and it remains the best-characterized MT polymerase, highly-conserved in all eukaryotes (Charrasse et al., 1996; Lee et al., 2001; Brouhard et al., 2008; Podolski et al., 2014). Affinity of XMAP215 for the MT plus-end is unique compared to many +TIPs, in that its localization does not depend upon EB1 (Brouhard et al., 2008). Rather, XMAP215 localizes to the far-distal plus-end, beyond EB1 (Maurer et al., 2014). While XMAP215 has been well-characterized *in vitro*, only a handful of studies have examined its function *in vivo*.

Initial studies of XMAP215-family members using neuronal cultures have confirmed its canonical role as a MT polymerase in neurons (van der Vaart et al., 2012; Lowery et al., 2013), but have also highlighted a novel growth cone-specific function for XMAP215 (Lowery et al., 2013). The mammalian ortholog, ch-TOG, was first shown to be important for promoting MT growth velocity, reducing MT catastrophe frequency in neurons, and enhancing axonal outgrowth (van der Vaart et al., 2012). However, in a separate study using *Xenopus laevis*, data gathered from growth cones after only partial inhibition of XMAP215 function revealed an unexpected function of this canonical +TIP. While partial knockdown did result in an increased rate of MT catastrophe (consistent with the ch-TOG study), MTs also exhibited a counterintuitive increase in MT plus-end velocities selective to growth cones (Lowery et al., 2013). Analysis by quantitative fluorescent speckle microscopy illustrated that differences in overall MT translocation/sliding, rather than MT polymerization, were a major contributor to the measured plus-end velocity change in the XMAP215 KD. In control growth cones, net translocation of MTs favored retrograde movement, attributed to frequent coupling of MTs to F-actin retrograde flow (Lowery et al., 2013). However, partial XMAP215 knockdown tipped the scales in favor of anterograde MT translocation. This finding indicated a previously undescribed role for XMAP215 in affecting MT translocation within the growth cone, possibly by mediating F-actin-MT crosslinking events. The balance of anterograde MT polymerization and MT sliding behaviors vs. the “drag” elicited by coupling to F-actin retrograde flow is thought to be instrumental in neurite and axon extension (Schaefer et al., 2002, 2008; Myers et al., 2006; Lee and Suter, 2008; Lu et al., 2013, 2015). Spatial regulation of MT polymer movements also likely contributes to axon guidance as well, although this remains to be explicitly shown. It is not entirely clear whether the role of XMAP215 in affecting MT sliding is direct or through additional intermediates, given that the *Drosophila* XMAP215 ortholog, *msps*, shows a genetic interaction with CLASP (Lowery et al., 2010), which is known to mediate MT-F-actin crosslinking (see above).

In fact, XMAP215 originally emerged as a prime candidate for modulating axon guidance when dual genetic and proteomic screens to identify CLASP interactors in *Drosophila* revealed that *msps* genetically antagonizes CLASP function and contributes to axon guidance in the embryonic CNS (Lowery et al., 2010). Overexpression of *msps* was also able to ameliorate a peripheral motor neuron “bypass” phenotype seen in *Abl* gain-of-function mutants (Lowery et al., 2010), further substantiating the role of an XMAP215-family member in axon guidance. However, only this single genetic study has thus far demonstrated a possible connection between XMAP215 and axon guidance. Accordingly, there is still an enduring motive to further define the cell biological functions of XMAP215 in growth cones, and deduce how these may ultimately mediate steering mechanisms.

### Transforming Acidic Coiled Coil (TACC3)

An ortholog to TACC3, member of the Transforming Acidic Coiled Coil (TACC) family (Piekorz et al., 2002; Aitola et al., 2003; Gergely et al., 2003), was similarly identified alongside XMAP215 in a screen for genetic interactors of CLASP in *Drosophila* (Long et al., 2013). Prior to this finding, the cytoskeletal functions of TACC3 were well-characterized primarily at the centrosome and mitotic spindle, where TACC3 is necessary for XMAP215 recruitment and astral and spindle MT elongation (Lee et al., 2001; Peset and Vernos, 2008; Lioutas and Vernos, 2013; Thakur et al., 2014). Thus, on one hand, the appearance of TACC3 in an interactome with XMAP215 was not entirely unintuitive. However, TACC3’s functions at the mitotic spindle were not specifically attributed to any plus-end regulatory capacity, and its presence at MT nucleation sites could not justify any function in guidance or outgrowth of post-mitotic neurons. Nonetheless, a subsequent examination confirmed that knocking down TACC3 in *Xenopus laevis* could drive significant decrease in axonal outgrowth length *in vitro* (Nwagbara et al., 2014). In live cells, TACC3 was shown to localize to the far-distal MT plus-end in embryonic neurons, where it co-localizes with XMAP215. Manipulation of TACC3 appeared to govern the fluorescent intensity of XMAP215 comets, suggesting that TACC3 recruits XMAP215 to the distal plus-ends of interphase MTs. It is likely this recruitment of XMAP215 that drives MT plus-end velocities; thus, TACC3’s function at the MT plus-end may be similar to its role at the mitotic spindle. Further studies will be useful to see if this XMAP215 recruitment can be spatially-regulated or modified downstream of signaling factors, as this could emerge as a potent mechanism to drive rapid MT extension, perhaps towards a chemoattractive gradient.

### End-binding (EBs)

The majority of +TIPs, including CLASP and APC, as well as others described below (the Spectraplakins, and the Navigators), do not actually bind to the MT plus-end or MT lattice directly. Instead, they localize via domains that target to plus-end scaffold proteins known as the end-binding (EB) proteins (Akhmanova and Steinmetz, 2008; Honnappa et al., 2009). It is worth noting that much of the MT plus-end dynamics studies that are so integral to cell biological ventures today are made possible by tracking and analysis of fluorescent EB1/3 constructs. This technique was first employed to track live, forward movement of MT polymerization in a study that examined MT directionality in various compartments of the young neuron, including the cell body, dendrites, and growth cone (Stepanova et al., 2003). Thus, EB1/3 association to the plus-end, which was confirmed to occur autonomously (Bieling et al., 2007), is crucial for many other +TIP interactions. For that reason alone, their role in axon guidance must be critical, albeit somewhat difficult to tease out from the complex of proteins that associate with them.

Another additional point of EB plus-end function comes from its interaction with drebrin (Geraldo et al., 2008), an actin binding protein. In immunoprecipitation assays, EB3 but not EB1, can immunoprecipitate drebrin from the growth cone cytosol. Drebrin shows strong immunolabeling with F-actin bundles in the proximal regions of filopodia. Colocalization of the two proteins appeared to occur when the distal tip of pioneer MTs came in contact with these F-actin bundles. The functional outcome of this interaction was tested with expression of a portion of EB3 that binds drebrin but not MTs; notably, these neurons were unable to extend growth cones. When drebrin was depleted, growth cones demonstrated abnormal MT bundling in the central domain, which prevented MT protrusion into filopodia. Together, these data demonstrate a drebrin–EB3, interaction is essential for MT extension into filopodia (Geraldo et al., 2008), which may be pivotal for interaction with localized signaling cascades.

Interestingly, classical or structural MAPs (MAP1B, Tau), which do not strongly associate the plus-end, can function within neurons to compete off or sequester EB1/3 proteins from the plus-end (Tortosa et al., 2013; Sayas and Avila, 2014). This grants these lattice-binding proteins the capacity to modulate an aspect of +TIP localization. Given that MAP1B and Tau association to the MT lattice are, in turn, regulated by GSK3 (Lovestone et al., 1994; Goold et al., 1999; Owen and Gordon-Weeks, 2003), this provides an additional opportunity for signaling cascades to modulate essentially all +TIPs simultaneously via their dependence on EB1.

### Cytoplasmic Linker Protein (CLIP-170)

Cytoplasmic Linker Protein (CLIP)-170, initially named for both its size and an association to cytoplasmic endocytic vesicles (Pierre et al., 1992), offers a myriad of potential signaling interactions, though precisely how these may function in axon guidance is still unfolding. Genetically and proteomically, CLIP-170 interacts with both CLASP and IQGAP (Fukata et al., 2002; Galjart, 2005), an actin-modulator downstream of Cdc42 and Rac1 (Swiech et al., 2011). This IQGAP-CLIP-170 interaction, in tandem with interactions with dynamic MTs, has been demonstrated to regulate actin during dendritic arborization (Swiech et al., 2011). While such a relationship has not been demonstrated explicitly during axon guidance, there have been several insights that point towards CLIP-170 having a role in F-actin remodeling in the growth cone (Figure 3).

During axon specification, inhibition of CLIP-170 was sufficient to inhibit axon formation (Neukirchen and Bradke, 2011). Likewise, overexpression could prompt the formation of multiple axons, highlighting a role for CLIP-170 in axon specification. The ability to accomplish this was attributed to a role for CLIP-170 in very modest stabilization of MTs, and specifically, a capacity to marginally increase MT rescue behaviors. This slight increase in stability facilitated the “push” of MTs to enter into the growth cone periphery. Actin phenotypes were also noted in growth cones that expressed a dominant negative CLIP-170, in that actin arcs appeared more robust and less penetrable by central MTs. Actin arc formation and trapping of MTs in the central zone in absence of CLIP-170 could be rescued with a brief treatment of actin depolymerizing drug, upon which axon elongation could resume (Neukirchen and Bradke, 2011).

Additionally, another examination of CLIP-170 in the axon showed that expressing a dominant negative version of CLIP-170 counterintuitively increased the growth rate and total growth distance of EB1-GFP comets (Stepanova et al., 2010), which could indicate changes in MT translocation (similar to those demonstrated with XMAP215) suggesting a decrease in transient coupling to F-actin retrograde flow. Given that CLIP-170 and CLASP interact (Akhmanova et al., 2001; Drabek et al., 2006), and CLASP is capable of performing just such a MT-actin crosslink (Drabek et al., 2006; Tsvetkov et al., 2007; Engel et al., 2014), it will be crucial to investigate the molecular underpinnings of a putative MT-actin link. While it may occur through CLASP, it is possible that CLIP-170 is also capable of this sort of crosslink independently of CLASP, perhaps through its interactions with IQGAP.

It is also worthy of consideration that while CLIP-170 is certainly expressed in early neural development, some researchers believe its role to be somewhat inconclusive (Beaven et al., 2015); their findings suggest that CLIP-170 may not factor into growth cone guidance significantly, as its plus-end binding in the nervous system, at least within *Drosophila*, appears greatly diminished compared to other cell types (Beaven et al., 2015). If this does hold true, it would be consistent with a number of other cases where +TIPs function differently in the neuron compared to other cell types (Stepanova et al., 2003; Lowery et al., 2013), emphasizing a continued need to examine +TIP function in the nervous system both in the context of the previous cell biological literature, as well as in its own right.

### Spectraplakins

The Spectraplakins demonstrate another family of +TIPs that are simultaneously able to bind MTs and F-actin (Leung et al., 1999; Fuchs and Karakesisoglou, 2001; Suozzi et al., 2012), and this F-actin-MT crosslinking is essential for axon extension (Lee and Kolodziej, 2002). ACF7, a Spectraplakin denoted Short-stop (Shot) in *Drosophila*, demonstrates an explicit axon guidance phenotype, in that deletion leads to motor axons that “stop-short” of their destinations (Vactor et al., 1993) and show defects in MT bundling (Alves-Silva et al., 2012). Similar to CLASP, Shot was shown to demonstrate disparate MT-binding mechanisms: one that allows lattice-binding, thus inducing bundling and MT stabilization, in addition to an EB1-mediated plus-end binding, which promotes axonal outgrowth (Alves-Silva et al., 2012).

While phosphorylation of Shot/ACF7 has not been examined in a neural context, ACF7 was shown to undergo phosphorylation by GSK3 in skin stem cells. This phosphorylation could inhibit MT-binding and disrupt polarized migration (Wu et al., 2011), a process that (like axon guidance) relies on spatiotemporally-regulated signaling and cytoskeletal dynamics (Horwitz and Webb, 2003; Wittmann and Waterman-Storer, 2005; Machacek et al., 2009). This study also demonstrated two sites of ACF7 phosphorylation by GSK3, and both were able to reduce MT-binding affinity (Wu et al., 2011), demonstrating yet another CLASP-like opportunity for a “sweet spot” of modulation, which may balance plus-end binding, lattice-interactions, dynamic-instability, and thus, axon-elongation.

An alternate or complementary means to explain a role for ACF7/Shot in axonal pathfinding was demonstrated by a link to Dual Leucine-zipper bearing Kinase (DLK; Valakh et al., 2013), which is part of a pathway that is crucial in normal apoptotic neuronal “pruning” and in axon regeneration (Tedeschi and Bradke, 2013; Chen et al., 2014a). RNAi against Shot, as well as a separate, hypomorphic Shot mutant, both demonstrated an “overgrown” synaptic phenotype, mediated by over-activation of DLK (Valakh et al., 2013). DLK activation has been shown to occur in response to cytoskeletal destabilization (Valakh et al., 2015). Therefore, either or both of these effects of Shot manipulation, the direct MT-actin perturbation or the downstream activation of DLK, may play into its deficits in pathfinding, a consideration that once again highlights the complex interplays that are required during cytoskeletal regulation.

### Navigators

Another class of +TIPs, the candidly-christened Neuron Navigators, were implicated in axonal pathfinding long before they were at all associated with MT plus-ends. Genetic screening in *C. elegans* first identified *unc-53* as critical for both epithelial and axonal guidance; mutant alleles of the gene demonstrated posterior body malformation, in addition to pronounced defects in mechanosensory neuron turning (Hedgecock et al., 1987). Subsequent searches in human, rat, and mouse identified a conserved family of genes, the Neuron Navigators (*NAV1*, *NAV2*, and *NAV3*), which coded for variations of a Calponin homology domain-containing protein which also featured putative actin-binding, signal transduction, and coiled-coil regions (Ishiguro et al., 2002; Maes et al., 2002; Merrill et al., 2002; Stringham et al., 2002; Martínez-López et al., 2005).

While genetic interaction data suggested the NAVs might act as intermediates between extracellular guidance cues and cytoskeletal machinery in axon guidance, especially when considered in tandem with their potential actin and signaling interactions, possible mechanisms were unclear. Localization data was inconsistent. Fluorescent-labeled homologs of NAV2 demonstrated cytosolic and nuclear localization (Ishiguro et al., 2002), whereas NAV3 appeared strictly nuclear (Coy et al., 2002). Intriguingly then, NAV1, which lacked the actin binding regions contained in the other paralogs, was shown to localize strongly to MT plus-ends, via a MT binding domain (MBD; Martínez-López et al., 2005). This localization shared comet-like morphology demonstrated by EB1. The capacity for NAV1 to act as a +TIP was thought to be unique, as initial efforts did not show plus-end localization of NAV2, despite strong conservation of the MBD domain in all Navigators (Muley et al., 2008). However, data gathered from later rounds of live imaging and immunofluorescence with new antibodies finally offered some evidence that NAV1, NAV2, and NAV3 may be true +TIPs, which could compete for plus-end localization with CLIP-170, attributable to an EB1-dependent binding mechanism (van Haren et al., 2009).

Cell biological studies of NAVs indicate that their role in axon pathfinding may stem from an ability to affect cellular-process organization. Compared to EB1, the Navigators exhibited a preferential localization at MT plus-ends in the periphery in multiple cell types, akin to that of CLASP (Martínez-López et al., 2005; Muley et al., 2008; van Haren et al., 2009). NAV1 specifically showed a pronounced enrichment in the peripheral zone of growth cones, though other NAVs were not tested (Martínez-López et al., 2005). However, overexpression of NAVs in non-neural cells induced MT bundling in neurite-like extensions, which were reported to resemble paused growth cones. This MT bundling appeared to occur with MT stabilization, as bundles colocalized strongly with acetylated-tubulin enrichment (Martínez-López et al., 2005; van Haren et al., 2009).

This ability to reorganize MTs is more poignant when coupled with the capacity of NAVs to interact with actin and mediate signaling cues. Early studies demonstrated that neurons transfected with NAV1 RNAi were less responsive to attractive Netrin signaling. Separately, the CH-domain within NAV2 *C. elegans* homolog was shown to directly interact with Abelson Interactor (ABI-1), an Abl substrate which functions in WAVE-complex mediated actin remodeling (Schmidt et al., 2009). The NAV2 homolog in *Drosophila* (Sickie) was suggested to promote axon growth by functioning in a non-canonical Rac GTPase pathway which ultimately facilitates Cofilin activation, providing an F-actin “recycling” mechanism to balance forward polymerization (Abe et al., 2014). To add another layer of intricacy, the MT plus-end localization of NAV1 was shown to be instrumental in enhancing the activity of TRIO, a Rho guanine nucleotide exchange factor which activates Rac1 and RhoG (van Haren et al., 2014); providing yet another platform for NAVs to influence actin remodeling within the growth cone.

## Conclusion

The growth cone is able to correctly maneuver through a myriad of extracellular cues, leading its axon accurately through the developing nervous system, turning in response to attractive or repulsive stimuli, and halting when it has arrived at the correct destination. The mechanisms by which this responsive cytoskeletal machine is able to detect and then translate numerous guidance signals are largely unknown. Here, we have illustrated how a subset of +TIPs can interact at the interface of these guidance cues to influence cytoskeletal behaviors. A striking theme behind the +TIPs that are characterized thus far in axon elongation and guidance, beyond their ability to undergo phosphorylation downstream of guidance molecules, is a strongly-shared ability to participate in transient MT-F-actin crosslinks or directly modulate F-actin behaviors (Figure 3). This ability offers a “missing link” of sorts, between signaling cascades, selective MT stabilization, and interactions that could then potentially impact filopodial and adhesion dynamics. Thus, cross-linking +TIPs can offer a feasible opportunity for mechanical translation of guidance molecules into axonal outgrowth and turning behaviors.

### Stories in Progress

It is almost certain that the relatively small list of +TIP modulators of axon guidance that we describe in this review will not be conclusive. We have neglected, for instance, the kinesins, whose roles and identities have expanded as they are seen increasingly to impact MT behaviors. Several kinesin family members interact with EB proteins (Lee et al., 2008; Gumy et al., 2013; Chen et al., 2014b) and demonstrate an effect on axon outgrowth through these +TIP interactions (Gumy et al., 2013). Additionally, kinesin-5 and kinesin-12 families have also shown commanding involvement in growth cone turning (Baas, 1998; Myers and Baas, 2007; Nadar et al., 2008; Liu et al., 2010), though whether an EB interaction is involved in their localization in this context is not known. Separately, CLIP-115 and CLIP-190 have been investigated in many shared examinations with CLIP-170 (Hoogenraad et al., 2000; Akhmanova et al., 2001; Stepanova et al., 2003; Neukirchen and Bradke, 2011; Beaven et al., 2015), but their individual roles in the growth cone are less well-clarified, and may emerge with time. As new +TIPs are frequently being established in a number of systems, it will be necessary to begin to not only consider their individual interactions in axon guidance, but also to examine their interplay with one another.

Additionally, we have described how numerous +TIPs are phosphorylated downstream of signaling molecules. However, it is clear that our current knowledge of these interactions is over-simplified. For example, a number of GSK3 substrates must first be phosphorylated by priming kinases, and these priming events have been shown to affect signal transduction within the growth cone (Uchida et al., 2005; Cole et al., 2006; Hida et al., 2012). Thus, phosphorylation by priming kinases may offer an additional layer of regulation for +TIP-MT-actin interactions. Indeed, phosphorylation of CLASP2 by CDK has been indicated to precede GSK3 activity and consequently regulate plus-end binding during mitosis (Kumar et al., 2012). Determining how multiple signaling cascades and kinase activities can be integrated constructively to designate a +TIP’s localization and behavior may be considered an ultimate pursuit within the field.

### Future Directions

Previous cell biological examinations of growth cone behavior and MT dynamics have relied on whole cell knockdown and overexpression of singular +TIPs. While these are not without merit, it is reasonable to imagine that these treatments can elicit misleading or heavy-handed compensatory phenotypes or unintended consequences, especially in the diverse cytoskeletal structures represented within the neuron. Micropipette or caged delivery of guidance molecules may be an enticing alternative to induce subtle, localized +TIP modulations, at least where we are appropriately familiar with their downstream effectors. But new options may emerge as photo-manipulatable (i.e., LOV, KillerRed, or SuperNova tagged) proteins are increasingly utilized, which would allow more targeted study of +TIP function (Bulina et al., 2006; Wu et al., 2009; Takemoto et al., 2013). In the guided growth cone, +TIPs must be regulated in a spatially and temporally-restricted manner. An ability to locally activate a +TIP phosphomimetic could allow a more physiologically-relevant way to recapitulate this, enabling investigation of perhaps one of the most pressing questions—how localized +TIP manipulation might induce changes in growth cone turning.

Efforts should also be made to translate what we know of axon guidance *in vitro*, from the familiarity of our substrate-coated coverglass, to 3-D substrates and haptotaxis gradients, as both are capable of inducing significant cytoskeletal remodeling (Santiago-Medina et al., 2015). Findings within these systems may challenge our current models of guidance and MT regulation. There is also the consideration, however, as microscopy evolves to become faster, finer, and brighter, that many systems are already well-situated for *in vivo* work. The ability to track individual growth cones in remarkable resolution through living brain tissue in zebrafish (St John et al., 2013) and frog (Leung and Holt, 2012) has allowed intricate investigations beyond what is possible with stripe assays and chemotaxis gradients. Additionally, MT plus-end dynamics have recently been imaged *in vivo* in mouse peripheral nerves (Kleele et al., 2014). Where* in vivo* imaging may currently fail to illustrate the finer mechanistic associations between MT plus-ends, F-actin, and assorted +TIPs, a nod must be given to critical advances in super resolution techniques (PALM, STORM, STED). As so many of the interactions on the MT plus-end occur below the diffraction limit of standard confocal microscopy, our current understanding of +TIP interactions is based predominantly on their biochemical interactions. Using super resolution microscopy, in addition to techniques such as FRET analyses, may allow us to model the plus-end scaffold in intricate detail, as has been accomplished with other cellular nanostructures (e.g., Kanchanawong et al., 2010). All of these emerging methodologies will provide new mechanistic insights into how the advance, retraction, and turning of the growth cone vehicle can be orchestrated during axon guidance, and how these behaviors are organized downstream of extracellular cues. Central to this, +TIPs have emerged as molecular tour guides that can inform and direct these axonal behaviors, by modulating their interactions with both actin and MT cytoskeletons in response to signaling cascades. It is evident, then, that future works that expand the breadth and depth of +TIP identification, function, and regulation will be instrumental to our understanding of axon guidance behaviors.

## Conflict of Interest Statement

The authors declare that the research was conducted in the absence of any commercial or financial relationships that could be construed as a potential conflict of interest.
